# Modulation of Wnt/Beta-Catenin Pathway by Major Dietary Phytochemicals Against Breast Cancer Development

**DOI:** 10.3390/biology14020194

**Published:** 2025-02-13

**Authors:** Noah Lieb, Annalisa Tran, Martha Torres, Ajay Bommareddy

**Affiliations:** Department of Biomedical Science, Charles E. Schmidt College of Medicine, Florida Atlantic University, 777 Glades Road, Boca Raton, FL 33431, USA

**Keywords:** breast cancer, DATS, resveratrol, capsaicin, EGCG, curcumin, BITC, naringin, quercetin, berberine, phytochemicals

## Abstract

Breast cancer is one of the most commonly diagnosed cancers and is the second leading cause of cancer-related deaths among women in the United States. Despite a decrease in mortality associated with breast cancer, there has been a steady increase in its incidence. Development of the mammary gland is normally regulated by such pathways including Wnt, Hedgehog, estrogen and Notch signaling. However, the deregulation of these pathways gives rise to tumor development, and upregulated Wnt activity along with high levels of beta-catenin is correlated with poor prognosis of breast cancer. In addition, beta-catenin-dependent Wnt signaling is enriched in triple-negative breast cancers and is associated with reduced overall survival in breast cancer patients. Various studies have investigated the ability of naturally occurring plant-based agents to reduce incidence and morbidity of breast cancer by regulating critical cell survival pathways to reverse or inhibit the occurrence of clinical disease. The present review focuses on summarizing the role of commonly consumed dietary phytochemicals and their role in regulating Wnt/β-catenin pathway against the development of breast cancer.

## Simple Summary

In the normal development of mammary gland development, Wnt signaling plays a vital role in maintaining normal function and homeostasis. However, dysregulation of the pathway, including increased Wnt activity and high levels of beta-catenin, are known to be associated with the development of different cancers including breast cancer, particularly triple-negative breast cancer. Owing to fewer treatment options available for treating advanced breast cancers, studies have explored the utility of naturally occurring phytochemicals as alternate preventive/treatment approaches. This review summarizes major dietary phytochemicals such as curcumin, capsaicin, tea polyphenols, garlic compounds, naringin, benzyl isothiocyanate, resveratrol, berberine, and quercetin that are widely consumed worldwide and their ability to modulate WNT/Beta-Catenin pathway to lower the burden of breast cancer development.

## 1. Introduction

Breast cancer remains one of the most commonly diagnosed cancers in the world. It is the second leading cause of cancer death in women, and in the United States alone the number of new breast cancer cases in 2024 are estimated to be 310,720, claiming more than 42,250 lives in the country each year [1]. Despite a steady decline in mortality at a rate of 1% from 2013 to 2019, incidence rates of female breast cancer have been slowly increasing by about 0.6% per year since the mid-2000s. The increase in incidence is primarily attributed to a decreasing fertility rate and increasing obesity. Being a complex malignancy, breast cancer, particularly the hormonal independent type is one of the most dreadful diseases with an increased risk of metastasis and relapse. Molecular mechanisms underlying the onset and progression of breast cancer are not fully understood, but various endogenous and exogenous factors are implicit with its development. Specifically, race, diet, estrogen secretion and metabolism, and other known risk factors such as family history, early menarche, and late menopause are responsible for the occurrence of breast cancer. Because some of these risk factors are not easily modifiable (e.g., genetic predisposition), other strategies for reduction in the breast cancer risk are desirable. Localized early-stage breast tumors are managed by surgery, radiation and estrogen-depleting therapy. However, once breast cancer progresses and metastasis occurs, they rarely respond to treatment. Selective estrogen-receptor (ER) modulators (e.g., tamoxifen) appear promising for prevention of breast cancer, but this strategy is largely ineffective against triple-negative breast cancers (TNBC). Compared to the other types of invasive breast cancer, TNBC has fewer treatment options. Hormonal therapy and anti-HER2 drugs are not effective for women with TNBC and hence chemotherapy or immunotherapy is used, often resulting in severe side effects. Chemotherapy, while effective in the treatment of TNBC, poses significant toxicity and often presents drug resistance. Similarly, the practice of immunotherapy is still in its early stages and clinical trials show that the effectiveness could vary from person to person and also depends on the staging of TNBC. The presence of tumor infiltrating lymphocytes, particularly T cells, in the tumor microenvironment predicts the response to immunotherapies and the tumors are considered immunoreactive if abundant T cell are present, while a lack of T cells indicates non-responsive tumors. In addition, the response is also affected by factors including the person, internal environment, etc., which are important to consider when assessing tumor-promoting and tumor-suppressing immune factors. Therefore, the development of agents that are safe and can delay the onset and/or progression of breast cancer, particularly TNBC is highly desirable and could have a significant impact on disease-related cost, morbidity, and mortality for a large segment of the population [2,3,4,5]. To that end, studies have explored the usage of naturally occurring phytochemicals along with synthetic agents to prevent, reduce, or even reverse the process of carcinogenesis. This review aims to critically appraise the current knowledge on the role of important dietary phytochemicals including Capsaicin, Curcumin, epigallo catechin gallate, diallyl trisulfide, Naringin, Benzyl isothiocyanate, resveratrol, Berberine, and Quercetin and their ability to modulate the Wnt/β-catenin pathway in breast cancer development. By focusing on the role of these natural products, we seek to enrich our current understanding of their involvement in breast cancer prevention and treatment. The information compiled could serve as a guide for future directions and offer insights for the development of diverse therapeutic regimens for tackling breast cancer. In this review, we compiled information by performing a literature search using the keywords “Beta-catenin pathway”, “Breast cancer”, and “Capsaicin, Curcumin, Berberine, Resveratrol, etc.” in PubMed and Google scholar until December 2024.

## 2. Wnt/β-Catenin Pathway

**Figure 1:**
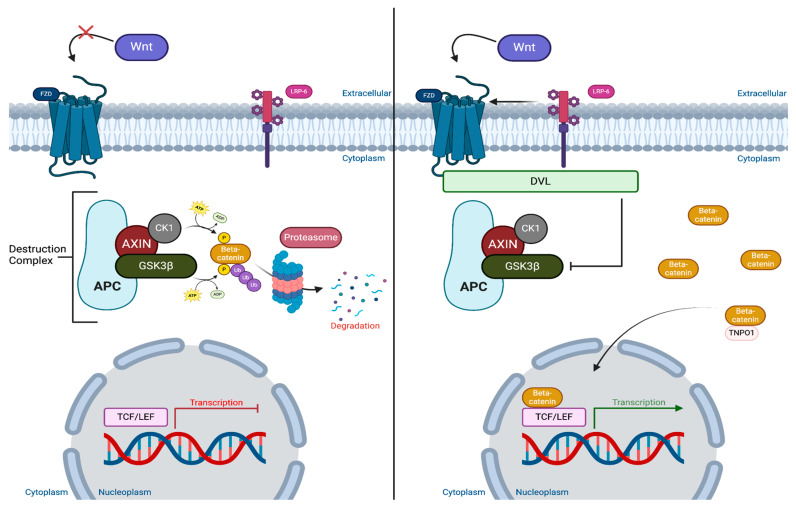
Wnt/β-catenin pathway: The canonical Wnt/β-catenin pathway has dual functionality whether a Wnt ligand is absent (**Left**) or present (**Right**). Without Wnt (**Left**) the destruction complex (DC), comprising axis inhibition protein (Axin), adenomatous polyposis coli (APC), casein kinase 1 (CK1), and glycogen synthase kinase 3β (GSK3β), is active. Axin and APC are scaffolding proteins that hold CK1 and GSK3β so that they could phosphorylate β-catenin. Once phosphorylated, β-catenin is marked for ubiquitination and proteasomal degradation. The result is low concentrations of cytoplasmic β-catenin and downregulation of transcription. Alternatively, when a Wnt ligand is present (**Right**), the Fzd receptor activates creating the multi-protein complex of Wnt, Fzd, and lipoprotein-related-receptor protein 6 (LRP6). This transduces a signal to the intracellular protein disheveled (Dvl) and creates the signalosome (Wnt/Fzd/Lrp6/Dvl/Axin/APC/CK1/GSK3β). Signalosome formation inhibits GSK3β/CK1 phosphorylation of β-catenin which increases cytoplasmic concentration. This allows Kap-β2/Transportin-1 (TNPO1) to bind and transport β-catenin across the nuclear membrane where it activates transcription factors T cell factor/lymphoid enhancer factor (TCF/LEF) and upregulates downstream transcription.

The canonical Wnt/β-catenin pathway functions either in the presence or absence of the extracellular ligand Wnt [6]. Wnt proteins are a class of highly conserved hydrophobic units bearing a signature palmitoleic acid on a specific Ser residue (Ser42) [6]. When inactive, or without the presence of the Wnt ligand, assembly and operation of the multi-protein complex known as the destruction complex (DC) is active [7]. There are four proteins that comprise the DC that function to scaffold and interact with the second messenger β-catenin in the Wnt cascade. Axis inhibition protein (Axin) and adenomatous polyposis coli (APC) are also important scaffolding proteins that hold Casein kinase 1 (CK1) and glycogen synthase kinase 3β (GSK3β) in place. CK1 and GSK3β both phosphorylate β-catenin which marks it for ubiquitination via proteasome [7]. This lowers cytoplasmic concentrations of β-catenin and ultimately results in downregulation of downstream genes [8]. However, in the presence of the Wnt ligand the Wnt-receptor Frizzled (Fzd), a highly conserved G-protein coupled receptor (GPCR), binds via specific binding features of a Cys-rich extracellular domain [9]. The affinity between the Wnt ligand and Fzd is also correlated with the sequestering of lipoprotein receptor-related protein 6 (LRP6) to initiate the signaling cascade [6]. The signal is transduced across the phospholipid bilayer via Fzd to Disheveled (Dvl) protein following the formation of the Wnt-signalosome. Disheveled is composed of three domains found across all mammalian paralogs which are the N-terminal DIX domain, the central PDZ domain, and the C-terminal DEP domain [10]. Most notably, the ability of the DEP domain of Dvl to modulate the polymerization of DIX domains via domain swapping plays a critical role in β-catenin stabilization [11]. Once the Dvl dimer is associated with Fzd, two important molecules in the destruction complex—Axin and GSK3β—phosphorylate LRP6, halting the phosphorylation of β-catenin [12]. The result is an accumulation of cytoplasmic β-catenin, and eventual transport across the nuclear envelope by Kap-β2/Transportin-1 (TNPO1). Once present in the nuclear space, association with T cell factor/lymphoid enhancer factor (TCF/LEF) transcription factors is modulated [13]. The β-catenin bound TCF/LEF transcription factors result in the upregulation of downstream genes *c-myc*, *cyclin D1*, *mmp7*, *mmp9*, and *MDR1* which normally function in early embryogenesis, but have been linked to neoplasm formation [14]. The Wnt/β-catenin signaling pathway (developed by using Bio render) is presented in Figure 1. Defects or mutations associated with the degradation complex components could lead to inappropriate stabilization of β-catenin and WNt target gene expression in the absence of a Wnt stimulus. For example, genetic alterations in Axin cause instability in the destruction complex and release β-catenin, thereby blocking the negative feedback loop and Wnt pathway regulation. Mutations in Axin also could increase Wnt target gene transcriptions and contribute to cancerous growth and development.

## 3. Phytochemical Classification

Phytochemicals are the non-nutrient compounds that are derived from consumption of a plant-based diet. Studies have shown the importance of phytochemicals in lowering the burden of various chronic conditions including different types of cancer. They have been identified to hold properties including anti-inflammatory, anticarcinogenic, DNA repair mechanisms, antioxidant, antiangiogenic, etc., which reduce the burden of cancerous growth and development. While consumption of whole foods imparts the beneficial effects of phytochemicals due to the synergism/potentiation of their effects in the presence of other micronutrients, minerals, and vitamins, research has focused on elucidating the pharmacological effects of individual phytochemicals and relating their usefulness as therapeutic alternatives. The classification of phytochemicals depends on their chemical structure, biological properties, and their source. According to the chemical structure, they are classified as carotenoids, alkaloids, phenolics, and organosulfur compounds. In the current review, we discuss major dietary alkaloids (capsaicin, berberine), phenolics (naringin, resveratrol, curcumin, quercetin, EGCG), and organosulfur compounds (DATS, BITC) and their ability to modulate Wnt/β-catenin pathway in reducing breast cancer burden.

## 4. Capsaicin

**Figure 2:**
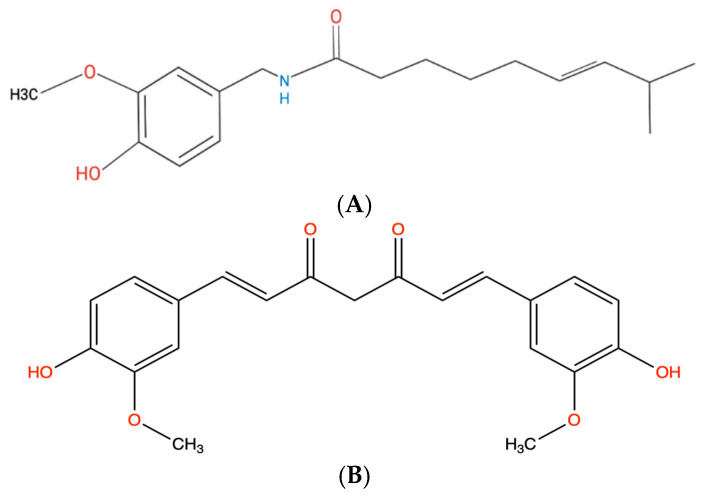

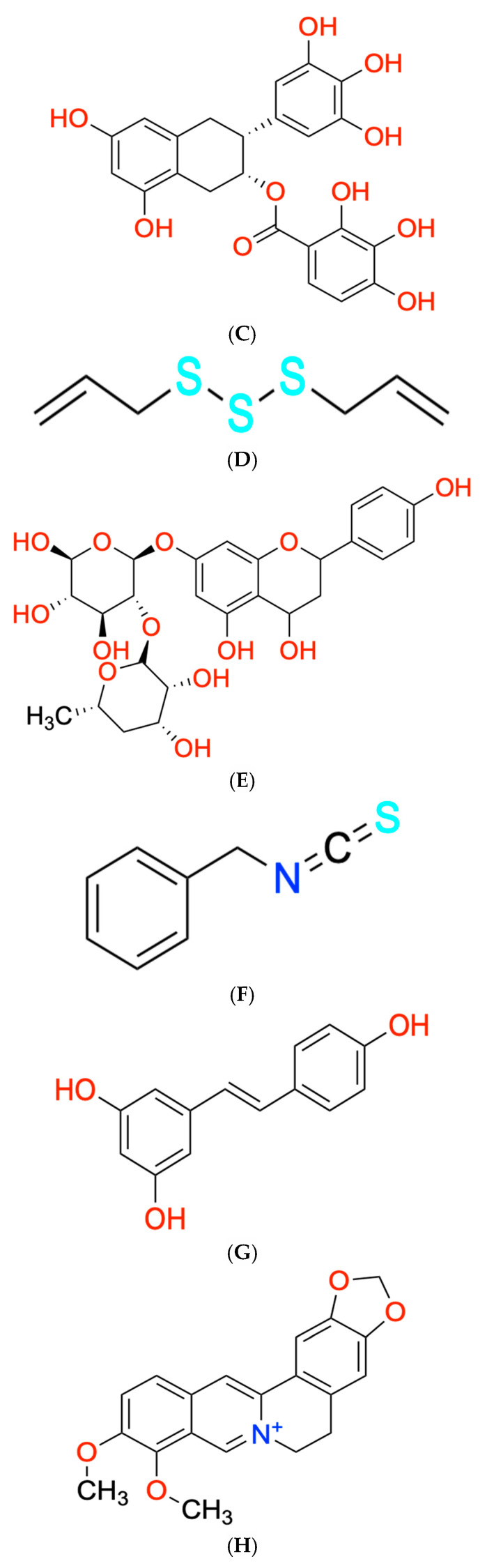

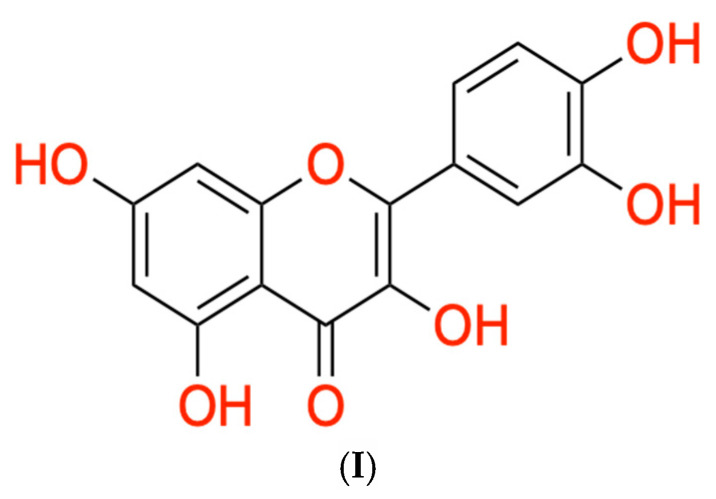
(**A**) Capsaicin. (**B**) Curcumin. (**C**) Epigallocatechin gallate (EGCG). (**D**) Diallyl trisulfide (DATS). (**E**) Naringin. (**F**). Benzyl Isothiocyanate (BITC). (**G**) Resveratrol. (**H**) Berberine. (**I**) Quercetin.

Chili peppers are comprised of numerous phytochemicals including flavonoids such as luteolin and apigenin which serve many antioxidant functions within the body [15]. However, there is another category of chemicals more notably identifiable for their inherent pungency called capsaicinoids. Within this family of chemicals, our most prominent member is capsaicin [16,17]. Capsaicin, ((E)-N-[(4-Hydroxy-3-methoxyphenyl)methyl]-8-methylnon-6-enamide) Figure 2A, is a member of the capsaicinoid/vanilloid family of chemicals and acts agonistically to TRPV1 receptors [17]. Separated into three sections, this phytochemical bears a vanillyl group and a hydrophobic side chain separated by an amide group. The signature vanillyl group provides H-bond donor/acceptor functionality to capsaicin, resulting in its agonistic binding [17]. Once ingested, this finicky chemical initiates a nociceptive response via agonistic binding of Transient Receptor Potential V1 (TRPV1) [18]. TRPV1 is a tetrameric ion channel with both its N-terminus and C-terminus kept intracellularly, which has an importantly high affinity for the vanillyl group of capsaicin [16]. Providing an extra kick to a dish is far from the only property that capsaicin exerts. Research has shown insight into the pharmacological relevance of this chemical in areas such as anti-inflammatory responses, analgesia, and chemoprotective influence amongst others [19]. In this review, we will limit our focus to capsaicin’s chemoprotective/anti-tumorigenic influence in multiple cell lines of human breast cancer via the Wnt/β-catenin pathway. Each cell line that was tested is a representative combination of the three widely accepted markers: estrogen receptor (ER), progesterone receptor (PR), and human epidermal growth factor receptor 2 (HER2) [20]. A study by Wu et al. [21] showed inhibition of the Wnt/β-catenin pathway in triple-negative breast cancer (TNBC) cell line MDA-MB-231 when treated with various concentrations of capsaicin (50, 100, and 200 μM) over a period of 24 h. Migration and cell viability was also tested with the MTT array discovering that cyclin-dependent kinase 8 (CDK8) was inhibited by capsaicin, reducing the viability of human breast cancer cells. The authors concluded that capsaicin inhibited breast cancer cell viability and migration by suppressing CDK8 expression and the PI3K/Akt/Wnt/β-catenin pathway [21]. Luminal A (MCF-7), Luminal B (BT-474), TNBC (MDA-MB-231), and HER2 (SKBR-3) cell lines were also tested at varying concentrations to assess cell cycle arrest and apoptosis activity following exposure to capsaicin. It was determined that at low concentrations (10^−5^ M) of capsaicin, ER^+^ cell lines (BT-474 and SKBR-3) had statistically significant percentages of cells arrested in the G_0_/G_1_ phases, and TNBC (MDA-MB-231) saw similar results at a higher concentration (2 × 10^−4^ M) within 24 h [22]. This demonstrated a particular affinity towards ER^+^ cell lines compared to TNBC variants. The exposure trials, however, showed significant percentages for BT-474, SKBR-3, and MDA-MB-231 at 67%, 46%, and 55%, respectively, following 2 × 10^−4^ M of capsaicin [22]. Utilizing similar concentrations and exposure times, it was observed that cell lines BT-474, SKBR-3, MDA-MB-231, and MCF-7 saw increases in the percentages of cells undergoing apoptosis when compared to the control [22]. While this is compelling evidence towards the anti-tumorigenic effects of capsaicin, one study demonstrated in vivo localization of radio metal-labeled capsaicin in breast cancer cells [23]. This was performed by administration of ^68^Ga-SCN-DOTA-Capsaicin into mouse models that possessed the MCF-7 and SKBR-3 cell lines. Results from this study using Positron Emission Tomography (PET) imaging showed notable uptake of ^68^Ga chelated SCN-DOTA-Capsaicin by the ER^+^ (MCF-7) cell lines [23]. Overall, evidence is overwhelmingly in favor of the oncostatic efficacy provided by the fruit from Capsicum Annuum, and further research continues to be performed.

## 5. Curcumin

Curcumin, [(1,7-bis(4-hydroxy-3-methoxyphenyl)-1,6-heptadiene-3,5-dione)] Figure 2B, is a diarylheptanoid that bears 2 O-methoxyphenol groups, a ɑ,β-unsaturated diketo-linker, and an enone moiety. The O-methoxyphenol functional group has proven critical for curcumin’s ability to perform ROS scavenging reactions [24]. Curcumin is a lipophilic polyphenol derived from *Curcuma longa Linn* (Turmeric) that has been utilized in traditional Indian medicine for thousands of years. There has been an accumulation of evidence supporting the pharmacological effects of curcumin (CUR) in cardioprotective, antioxidant, antibacterial, anti-inflammatory, and antitumor activities [25]. We will limit our discussion to the pharmacokinetic and pharmacodynamic properties of curcumin and its antitumor properties in breast cancer cell lines. One of the major cellular targets for CUR is the Wnt/β-catenin pathway which plays a well-documented role in early embryogenesis [6]. The canonical Wnt/β-catenin pathway is dependent on β-catenin as functionality is derived from the accumulation of β-catenin in the intracellular space, and in early development, Wnt/β-catenin modulates many aspects of differentiation, and proliferation [6]. However, interference and deregulation of the pathway are heavily associated with cancer cell proliferation as excess β-catenin migrates to the nuclear space. After infiltration of the nuclear envelope, the transcription of genes (e.g., *MYC and CCND1*) is initiated [8,26]. An in vitro study demonstrated CURs influence on downregulation of the aberrant Wnt/β-catenin pathway in breast cancer stem cells (Breast CSC) via inhibition of phosphorylation of GSK3β [27]. Phosphorylation of GSK3β at Ser^9^ by various kinases has been found to upregulate transcription of downstream genes in the Wnt/β-catenin pathway. Inhibition of GSK3β phosphorylation was confirmed via Western blot and reflected downregulation of the *MYC* gene [27]. In vivo and in vitro studies also documented a direct correlation between tested concentrations (10–30 μM) of CUR on apoptosis and inhibition of proliferation of various cultured breast cancer cell lines [28,29,30]. Evidence supports the linking of the suppression of cell cycle progression of MDA-MB-231 cells by CUR directly to the inhibition of the Wnt/β-catenin pathway [26]. Western blot analysis of MDA-MB-231 and MCF-7 cells treated with 20 μM CUR was found to have a 2-fold reduction in expression of β-catenin after 12 h. The MCF-7 cells also noticeably had increased expression of GSK3β at 4.5-fold [31]. Both cell lines also saw a similar correlation between CUR exposure and increased cell arrest in the G2/M phase as in the other studies. The outlook for the administration of curcuminoids, which include CUR and its analogs bisdemethoxycurcumin (BDMC) and demethoxycurcumin (DMC), for their antitumor capabilities is promising. There lies an overwhelming amount of literature that supports the effectiveness of curcuminoids against growth of human breast cancer cells [32].

## 6. Epigallocatechin Gallate

Extracted from the leaves of the *Camellia sinensis* plant, more commonly known as green tea, is one of the common catechins epigallocatechin gallate (EGCG), [(2R,3R)-5,7-dihydroxy-2-(3,4,5-trihydroxyphenyl)chromen-3-yl], 3,4,5-trihydroxybenzoate, Figure 2C, a catechin polyphenol derived from *Camellia sinensis*. This polyphenol bears a galloyl moiety which helps to improve binding affinity [33]. There are also numerous hydroxyl groups on the phenol rings that can facilitate hydrogen bonding with target receptors [33,34]. This phytochemical is a polyphenol ester derived from epigallocatechin and gallic acid that provides many well researched antioxidant properties [35]. A standard cup of green tea contains approximately 170 mg of EGCG per 2.5 g of green tea leaves steeped [36]. Once brewed and ingested, EGCG facilitates its antioxidant effects via agonistic interactions with trans-membrane receptor 67-kDA laminin receptor (67LR) [37]. In vivo activation of 67LR has been linked to downregulation of β-catenin expression in different cancers. Binding with the YIGSR peptide of the laminin β1 chain has been proven to inhibit the expression of β-catenin while antibody modulated blocking of 67LR revealed restoration in this process [38]. EGCG, bearing a similar motif to the YIGSR peptide, was found to have strong binding affinity for a 10-residue portion of human 67LR at residues 161–170 [39]. The introduction of peptide IPCNNKGAHS neutralized cell-surface binding and antineoplastic properties of EGCG. This was confirmed by the creation of the EGCG-LR161-170 protein complex on mass spectrometric analysis and SPR assay [39]. Studies were also conducted on TNBC cell line MDA-MB-231 to test the effects on cell viability and β-catenin expression in the presence of EGCG at varying concentrations [40]. Administration of various concentrations of EGCG for 24 h showed a dose-dependent decrease in MDA-MB-231 viability. Treatment of these cells also demonstrated a significant decrease in β-catenin expression [40]. Another study utilized high-performance liquid chromatography (HPLC) to quantify the concentration of active substituent EGCG in both matcha (powder of shade-grown tea leaves) and green tea. The samples were then administered to both TNBC and estrogen receptor positive (ER^+^) cell lines, and viability was calculated using ATP luminescence testing [41]. It was calculated that dose-responsive decrease in cell viability was statistically significant in doses of 180, 150, 120, and 90 μg/mL (all > ED_50_) in MDA-MB-231 (TNBC) cell lines. Repeat testing with MCF-7 (ER^+^) cell lines showed >ED_50_ for the same doses [41]. Results not only demonstrated the anti-tumor properties of EGCG, but also proposed the involvement of an estrogen receptor-independent pathway [41]. Along with the aforementioned downregulation of the Wnt/β-catenin pathway via 67LR that was demonstrated [38], it is also noted that EGCG may exert inhibitory effects on telomerase as well as catalytic gene production from *hTERT* in ER^+^ cell lines [42]. Presently, there are also highly supportive papers documenting the effects of quercetin, kaempferol, and myricetin (other polyphenols commonly found in *Camellia sinensis*) on breast cancer [43,44,45]. Kaempferol, specifically, was discovered to increase levels of caspase 9 (CASP9), induce apoptosis, and coordinate G2/M phase cell cycle arrest in TNBC [44]. Reports also link apoptotic rates of TNBC of myricetin via numerous pathways including PI3K/AKT/mTOR amongst others [45]. Overall, the evidence is overwhelmingly in favor of integrating EGCG treatment into oncological/clinical settings.

## 7. Diallyl Trisulfide (DATS)

Throughout centuries, *Allium sativum* L. (garlic) has been used by many different cultures around the world to treat a variety of ailments [46,47,48,49,50]. Diallyl trisulfide (DATS), (3-(prop-2-enyltrisulfanyl)prop-1-ene), Figure 2D, is an organosulfur compound, derived from *Allium sativum* L. It bears a trisulfide linkage flanked by allyl groups, forming unique chemical and pharmacological properties. It has been shown to exhibit inhibitory effects on estrogen receptor-α (ER-β) in various cell lines [47]. DATS exerts its antitumor effects by interfering with cancer cell growth, tumor metastasis, vascularization, and cell signaling pathways that regulate cell apoptosis and cancer cell mitosis [49]. Recent investigations have explored the role of DATS in regulating the activity of breast cancer stem cells and the underlying molecular mechanisms. In a study conducted by Li et al. [51], it was reported that DATS efficiently inhibited the viability of breast CSCs by reducing tumorsphere formation, decreasing the expression of breast CSCs markers (CD44, ALDH1A1, Nanog, and Oct4), and inhibited proliferation while inducing apoptosis as well as downregulating the activity of the Wnt/β-catenin pathway. Breast cancer cell lines MCF-7 and SUM159 were exposed to varying concentrations of DATS for 7 days in vitro which resulted in a significant reduction in the size and numbers of tumorspheres with increase in the concentration of DATS. DATS also decreased the mRNA levels of breast CSCs markers (CD44, ALDH1A1, Nanog, and Oct4) in both MCF-7 and SUM159 tumorspheres when compared to the control group. Furthermore, DATS decreased the levels of cell proliferation proteins PCNA and Cyclin D1 in both MCF-7 and SUM159 tumorspheres in a concentration dependent manner. DATS reduced the expression of anti-apoptosis protein Bcl-2 while the levels of pro-apoptosis protein Bax and caspases (Caspase 8, Cleaved-Caspase 9, and Cleaved-Caspase 3) increased significantly. These results revealed that DATS inhibited proliferation and induced apoptosis of breast CSCs. It also led to decreased p-GSK3β and increased GSK3β. It showed decreased β-catenin as well as downregulating the level of β-catenin downstream gene *c-Myc*, which suggested that DATS suppressed the activation of the Wnt/ β-catenin pathway. To further examine the role of the Wnt/β-catenin pathway in DATS’ effects on breast CSCs, LiCl, a GSK3β inhibitor, was utilized. Results showed that LiCl weakened DATS impact on p-GSK3β, β-catenin, *c-Myc*, and the expression of breast CSC markers (CD44 and ALDH1A1). Additionally, LiCl reversed DATS’ inhibition of proliferation and induction of apoptosis in breast CSCs. These findings suggest that Wnt/β-catenin activity is critical for DATS’ suppression of breast CSCs [51].

Various preclinical models including xenograft mouse models and chemically induced mammary cancer rat models were employed to assess the effectiveness of DATS against breast cancer development. For example, oral administration of DATS (0.9 mg/kg body weight) reduced the tumor volume of MCF-7 xenografts [52] but daily administration of DATS (25 to 50 mg/kg body weight) did not affect tumor burden but significantly reduced lung metastasis in MDA-MB-231 xenografts [53,54]. More recently, oral gavage of DATS (50 mg/kg body weight) 5 times a week for 10 weeks in a chemically induced breast cancer tumor model did not inhibit tumor growth, and the authors concluded that much higher doses of DATS would be required to achieve a significant reduction in tumor burden [55]. Bodyweight was not affected by the administration of DATS in the study [55]. It was concluded that reactive oxygen species (ROS) generation could play an important role in the efficacy of DATS against breast cancer development.

## 8. Naringin

Naringin, (3,5-Dihydroxy-4-[3-(4-hydroxyphenyl)propanoyl]phenyl 2-O-(6-deoxy-α-L-mannopyranosyl)-β-L-glucopyranoside), Figure 2E [56], a flavonoid that belongs to the flavanone subclass, is a natural glycoside present in many plant species, especially in citrus fruits such as grapefruits and oranges [56,57] and using its heterocyclic pyran ring it serves biological functions [56]. Over the years, numerous studies have highlighted naringin’s ability to modulate different signaling pathways and interact with diverse cell signaling modules, enabling it to mitigate inflammation, oxidative stress, metabolic syndromes, bone disorders, and cancer. This phytochemical has been shown to inhibit different types of cancer through growth suppression of malignant cells and induction of apoptosis [58].

Although multiple studies [59,60] have been published on the effects of naringin against breast cancer, only one investigation [61] has reported that naringin inhibits the growth potential of human TNBC cells by targeting the β-catenin pathway. Treatment of TNBC cell lines including MDA-MB-231, MDA-MB-468 and BT-549 with different concentrations of naringin significantly inhibited cell proliferation of the cells. Flow cytometry analysis of cell cycle revealed a significant increase in G1-phase cells in the naringin-treated group compared to control group, suggesting G1 cell cycle arrest by naringin in TNBC cells. To identify the cell apoptosis-related molecules involved in naringin-induced apoptosis, the human apoptosis antibody array was employed in MDA-MB-231 cells. The analysis showed that there was a significant overexpression of p21 and suppression of survivin in naringin-treated cells. These findings were further validated by Western blot and immunofluorescence analysis in both MDA-MB-231 and BT-549 cells. In the same study, inhibition of β-catenin activity was also confirmed by TOPFlash dual-luciferase reporter assay. To verify that inactivation of the β-catenin pathway is responsible for the antitumor activity of naringin, adenovirus over-expressing β-catenin or siRNA β-catenin was used. It was found that abrogating β-catenin by Ad-siRNA β-catenin led to the inhibition of cell proliferation in TNBC cells. It was also discovered that p21 and survivin were modulated by β-catenin in breast cancer cells. The overexpression of β-catenin induced the downregulation of p21 and upregulation of survivin, while specific knock out of β-catenin had the opposite effect. These results suggested that naringin exerted antitumor activity in TNBC cells by modulating β-catenin pathway. An MDA-MB-231 xenograft model was utilized to analyze the antitumor potential of naringin in vivo. The results showed significantly smaller tumor volumes and tumor weights in the naringin treated mice. Proliferation in tumor specimens decreased significantly, while markers of apoptosis in tumor specimens were increased in mice treated with naringin. The results verified the findings in vitro and validated that naringin significantly inhibited breast cancer development [61].

## 9. Benzyl Isothiocyanate (BITC)

BITC (Isothiocyanatomethyl) benzene, Figure 2F is one of the most common naturally occurring isothiocyanates present in various cruciferous vegetables including broccoli, water cress, etc., and has been shown to be effective against the development of various cancers in preclinical studies [62]. The antitumor properties stem from its Isothiocyanate group attached to the benzyl ring. The benzyl ring creates a lipophilic environment and, coupled with an electrophilic carbon on the Isothiocyanate group, allows for hydrolysis and reactive oxygen species formation [62]. Isothiocyanates including BITC are produced through hydrolysis of corresponding precursors (glucosinolates), catalyzed by myrosinase in response to pathogens and environmental changes influencing the plants [63].

As reviewed by Dinh et al. [62], the cancer preventive and anti-tumor properties of BITC have been well documented in 14 different cancers including breast cancer. BITC’s pharmacological properties in lowering cancer burden have been attributed to its ability to modulate important cell signaling pathways associated with cell proliferation, cell cycle arrest, apoptosis, ferroptosis, autophagy, angiogenesis, and metastasis. As discussed in the earlier sections, aberrant activation of Wnt/β-catenin can influence its downstream targets, including c-Myc and cyclin D1 which play a major role in cancer cell proliferation and growth. Studies show that BITC plays a major role in inhibiting epithelial–mesenchymal transition (EMT) by inducing E-cadherin levels and downregulating FOXQ1 levels in cellular and preclinical mice models [64]. FOXH1, another transcriptional factor of the Forehead-box family of transcription factors is known to positively regulate e-cadherin and activate the Wnt/β-catenin pathway and contribute to tumor progression. Liu et al., 2015 [65] showed that knockdown of FOXH1 augments BITC-mediated inhibition of cell proliferation and invasion in MDA-MB 231 and SUM 159 TNBC cells. In another study, it was also shown that BITC treatment inhibited breast cancer tumorigenesis in vitro and in vivo through suppression of the FOXOH-1-mediated Wnt/β-catenin pathway. Specifically, the authors showed that the expression of tumor promoters β-catenin and Wnt2 was inhibited in response to BITC treatment, while the downstream effectors of β-catenin such as cyclinD1 and c-Myc were downregulated. Furthermore, expression levels of APC and GSK-3 β were notably increased in cells treated with BITC compared to the control cells, which was also further evidenced by increased levels of APC in the tumor samples of the BITC treatment group. It was concluded that BITC’s antitumorigenic potential in mammary carcinoma may be mediated through the regulation of the Wnt/β-catenin pathway and the APC- GSK-3 β degradation complex [66].

## 10. Resveratrol

Resveratrol (3,5,4′-Trihydroxystilbene), Figure 2G [67], is a naturally occurring polyphenolic compound that is found in abundance in grapes, peanuts, berries, and red wine. Resveratrol has been extensively studied for its anti-inflammatory, anti-tumor, cardioprotective properties and its ability to increase the lifespan of mammals [67,68]. The chemotherapeutic and chemopreventive properties of resveratrol have been well established in preclinical models and the functionality of resveratrol comes from its hydrophobicity and stilbene structure [67]. Studies employing different in vitro and in vivo cancer models show that resveratrol targets the cyclooxygenase enzyme, involved in the generation of pro-inflammatory molecules that are involved in tumor growth, and downregulates the AKT, MAPK, and NF-κB signaling pathways to reduce tumorigenesis and inflammation [69].

CSCs, by undergoing differentiation and self-renewal, have the ability to generate cells with a distinct phenotype within the tumors. The Wnt signaling pathway along with Notch and Hedgehog are known to play a crucial role in the regulation of CSCs self-renewal and differentiation. A study by Fu et al. [70] showed that resveratrol suppresses the Wnt/β-catenin pathway in breast CSCs in vitro and xenograft breast tumors in vivo. Specifically, it was shown that resveratrol decreases the expression of β-catenin and cyclinD1 in CSCs and xenograft tumors. In addition, the authors also showed that resveratrol induced autophagy could also be mediated through suppression of the Wnt/β-catenin pathway. Over-expression of β-catenin by transient transfection and inhibition of autophagy using chloroquine (autophagy inhibitor) reduced the anti-tumor effects of resveratrol. The authors concluded that resveratrol inhibits breast CSCs growth and induces autophagy, at least partially, by downregulating the Wnt/β-catenin pathway [70]. A different study [71] that explored the antitumor properties of 3,5,4-trimethoxystilbene (MR-3), a naturally methoxylated derivative of resveratrol, revealed an increase in the expression of E-cadherin, an epithelial marker, and decreased the expression of mesenchymal markers including snail, slug, and vimentin. The study further showed that the suppression of EMT and further invasion of MCF-7 cells by MR-3 was mediated through the suppression and nuclear translocation of β-catenin and downregulation of its downstream target genes, *c-myc* and *cyclin D1*. In addition, MR-3 also restored GSK-3 β activity by inhibiting phosphorylation of Akt. Overall, the study concluded that MR-3’s role on its anti-invasive activity on MCF-7 cells was mediated through the suppression of EMT occurring through downregulation of PI3K/AKT signaling and subsequent β-catenin nuclear localization [71]. In a recent study, it was shown that resveratrol reverses the EMT properties of MCF-7/ADR (multi drug resistant breast cancer cells) by regulating the connection between SIRT1 and β-catenin pathway. Specifically, the authors identified that resveratrol reversed the EMT phenotypic characters of MCF-7/ADR cells by downregulating the expression levels of vimentin, N-cadherin, and β-catenin while upregulating the expression of SIRT1 (a negative regulator of WNT/β-catenin pathway). The role of SIRT1 in resveratrol-mediated anti-invasive effects was further confirmed by generating MCF7/ADR-shSIRT1 cells using shSIRT1 lentivirus, where it was shown that shSIRT1 treatment significantly reversed the inhibiting effects of resveratrol on MCF7/ADR cells. Furthermore, overexpression of SIRT1 established the inverse association of SIRT1 and β-catenin degradation by promoting its ubiquitin-mediated proteolysis in MCF-7/ADR cells. It was concluded that resveratrol reverses doxorubicin resistance by upregulating SIRT1 and negatively influencing the β-catenin pathway in MCF-7/ADR breast cancer cells [72].

## 11. Berberine

Berberine (5,6-dihydro-9,10-dimethoxybenzo[g]-1,3-benzodioxolo [5,6-a]quinolizinium), Figure 2H [73] is a tetra-substituted alkaloid bearing methylenedioxy groups at carbons 2 and 3 as well as methoxy groups at carbons 9 and 10. These groups, as well as having a high affinity for nucleophilic substitution about the 8th carbon, provide berberine with its functionality [73]. It is from plants such as *Berberis vulgaris*, *Berberis aristata*, Goldenseal, or Oregon grape and has been traditionally used in Chinese, Ayurvedic, and Native American herbal medicines [74,75]. There is evidence in the literature of its pharmacological applications against viral infections, inflammation, and tumorigenesis [76,77]. Previous research has shown the effect of berberine treatment against multiple cancers, such as colon cancer or gastric cancer [78,79]. Due to its applications against multiple cancers, there is a growing interest in studying its potential against breast cancer development. Berberine, as well as many of its derivatives and semi-synthetic analogs, have been investigated for their role in mammary tumor cells. These berberine derivatives are developed in a variety of methods. For instance, epiberberine (EBR) is an isomer of berberine (BBR), berberrubine (BRB) is developed from heating up berberine, and dihydroberberine (DBR) is formed when berberine undergoes hydrogenation [74]. Semi-synthetic analogs of berberine contain aromatic groups attached to the 13-position of the parent alkaloid skeleton and are given names such as NAX012, NAX014, NAX035, NAX053, NAX057, NAX060, NAX085, and NAX118 [75]. 

Berberine, its derivatives, and its analogs have been identified to induce apoptosis and inhibit the viability and migration activity of both human and canine mammary tumor cells [74,75]. As reviewed by Zhong et al. [80], BBR exhibits antitumor properties by interacting with various proteins and DNA sequences. For example, BBR was found to induce cell cycle arrest, reduce expression of cyclin D and cyclin E, and inhibit the AMPK pathway to induce apoptotic cell death in different breast cancer cells. Studies also suggest that berberine may play a role in Wnt/β-catenin signaling activity. When Western blot analyses were conducted to observe β-catenin protein expression in CF33 canine mammary tumor cells, it was observed that the total amount of β-catenin and the expression of active β-catenin decreased in CF33 cells treated with berberine analogs (NAX035, NAX053, NAX057, and NAX060) [75]. Similarly, epiberberine and berberrubine treatment downregulated β-catenin expression and upregulated GSK-3β expression in MDA-MB 231 and MCF-7 cells [74]. Further evidence of berberine’s effect on Wnt/β-catenin signaling activity was found in a study where zebrafish embryos with a reporter gene mCherry that is associated with the Wnt/β-catenin signaling pathway were treated with berberine and its analogs. Zebrafish Tg(7xTCF.Xlasiamois:nlsmCherry) and Tg(Hsa.CTGF:mCherry) transgenic lines were analyzed with fluorescent microscopy imaging, where greater fluorescence of the reporter protein mCherry indicated Wnt/β-catenin signaling activity [75]. Lower fluorescence was observed in Tg(7xTCF.Xlasiamois:nlsmCherry) reporter animals that received berberine, NAX035, NAX053, and NAX057 treatment [75]. This indicates that berberine and its analogs do play a role in modulating the Wnt/β-catenin signaling pathway and limit the proliferation and growth of mammary tumor cells. In addition, berberine and its derivatives have been observed to affect negative regulators of the Wnt/β-catenin pathway such as E-cadherin, which is a protein involved in maintaining cell–cell adhesion. Studies show that low levels of E-cadherin mimics Wnt signaling, allowing free β-catenin to associate with LEF-1, a transcription factor, in the nucleus and contribute to cancer cell growth [81]. E-cadherin levels were significantly elevated after treatment with berberrubine and epiberberine in MCF-7 cells [74]. Similarly, in MDA-MB-231 cells, E-cadherin levels were significantly elevated after DBR treatment [74]. Consequently, by enhancing E-cadherin levels, berberine and its derivatives inhibit Wnt signaling, limiting cancer cell growth. While these studies show the antitumor potential of BBR, much needs to be elucidated regarding its analogs and also the potential of BBR when used in combination with other existing chemotherapeutic agents/phytochemicals in reducing breast cancer burden.

## 12. Quercetin

Quercetin (3,3′,4′,5,7-pentahydroxyflavone), Figure 2I, a polyphenolic flavonoid, is derived from various forms of vegetation and fruits including apples, berries, and radish leaves. Its elaborate physicochemical and pharmacological properties are governed by the dihydroxy, o-dihydroxy, and phenolic groups present [82,83]. Studies identified the anti-cancer properties of quercetin in various cancer cell models, such as prostate, pancreatic, liver, and gastric cancers [19,83,84,85,86]. However, little has been elucidated about quercetin’s role in breast cancer. Some studies report that quercetin inhibits breast cancer cell growth, induces apoptosis, and causes change in the morphology of breast cancer cells [19,85]. For example, when treated with quercetin, the morphology of MDA-MB-231 and MDA-MB-468 cells changed from spindle-like fibroblast shapes to a rounded, cobblestone epithelial phenotype [19]. These results suggest that quercetin plays a role in cancer cell regulation. The mechanisms of how quercetin regulates cancer cell growth and shape are still unclear. Many studies observed possible pathways that quercetin influences, such as the Wnt/β-catenin signaling pathway. In 4T1 murine mammary cancer cells, Wnt/β-catenin signaling activity was measured using a luciferase assay and quercetin suppressed ~50% of basal level luciferase activity, indicating a suppression of Wnt/β-catenin signaling activity [85]. To determine how quercetin suppressed Wnt/β-catenin signaling activity, Western blot analyses were conducted and showed a reduction in β-catenin stabilization 1 h after quercetin treatment [85]. Similar results were observed in MDA-MB-231 cells treated with quercetin, where immunofluorescence assay revealed localization of β-catenin expression in the cytoplasm compared to the nucleus [19]. In the same study, when MDA-MB-231 cells were treated with quercetin, E-cadherin expression levels significantly increased compared to vehicle-treated cells. Further analysis revealed that E-cadherin mRNA expression and protein expression in MDA-MB-231 cells showed a nine-fold increase and ten-fold increase, respectively, after quercetin treatment [19]. Therefore, these findings suggest that quercetin influences β-catenin activity and also plays a role in regulating E-cadherin expression, which can inhibit the Wnt/β-catenin signaling pathway. Quercetin has also been observed to influence proteins that regulate the Wnt/β-catenin signaling pathway. Dickkopf (DKK) is a family of proteins that act as extracellular inhibitors of the Wnt/β-catenin signaling pathway [87,88]. In the classical pathway (Wnt/β-catenin), DKK proteins, specifically DKK1, by binding to LRP6 coreceptor with high affinity, impedes β-catenin-dependent Wnt signaling and is also involved in the formation of the complex of DKK1 with LRP5/6 and KrementFrizzled, resulting in β-catenin phosphorylation, preventing localization of β-catenin to the nucleus and inhibiting target gene expression related to cell cycle, tumor growth, and invasion. However, DKK1 has also been linked to the promotion of cancer, and the ability of DKK1 to function as a tumor suppressor or promoter depends on numerous factors including the type of cancer, heterogeneity within the tumor, and tumor microenvironment [89,90]. RT-PCR analysis of DKK1, DKK2, DKK3, and DKK4 in 4T1 murine mammary cancer cells showed that the expression levels of DKK1, 2, 3, and 4 increased in a dose-dependent manner, with the expression of DKK1 increasing more than 3-fold, the expression of DKK2 and 3 increasing about 2-fold, and DKK4 expression levels showing a nonsignificant increase [85]. The authors further confirmed the role of DKK1 on growth suppression of cells by using recombinant DKK1 and compared the results with cells treated with quercetin. Based on the results, the authors concluded the possibility of other antagonists involved in the regulation of Wnt/β-catenin and that the findings from the study show that the growth suppression and induction of apoptosis by quercetin-mediated murine mammary cancer cells is possibly mediated through DKK-dependent inhibition of the Wnt/β-catenin signaling pathway. 

## 13. Clinical Trials

While there are some clinical trials conducted on cancer patients using the phytochemicals reviewed in this paper, only a few clinical trials were conducted with breast cancer patients specifically, and even fewer trials that study how phytochemicals affect the proliferation of mammary tumors directly. A clinical trial, conducted by Saghatelyan et al. [91], involved 150 breast cancer patients receiving either intravenous curcumin in combination with paclitaxel, a commonly prescribed chemotherapeutic agent, or a placebo in combination with paclitaxel [92]. It was found that the percentage of patients with complete or partial reduction in tumor size was significantly higher in the group receiving curcumin + paclitaxel compared to the placebo + paclitaxel after 12 weeks of treatment [92]. In addition, after 3 months post-treatment, the percentage of patients exhibiting complete or partial reduction in tumor size remained significantly higher in those who received curcumin + paclitaxel than those who received placebo + paclitaxel [92]. Currently, there are other clinical trials that are ongoing to explore the efficacy of the discussed phytochemicals. For example, clinical trial NCT03980509 (ongoing) studying curcumin treatment in breast cancer patients aims to determine if curcumin can decrease tumor proliferation rate. Another, Trial NCT06355037, seeks to study the combination of dasatinib and quercetin treatment in preventing chemotherapy resistance in triple-negative breast cancer patients. A list of clinical trials with their outcome is presented in Table 1. Overall, a lack of breadth in comprehensive clinical trials exists due to the fewer preclinical studies on phytochemicals and their effects on breast cancer growth and development. There is a noticeable gap in the literature which warrants more investigations to determine if these phytochemicals are effective alternatives to chemotherapy and immunotherapy in breast cancer patients. 

## 14. Discussion

As discussed earlier in the paper, the protein kinase GSK3β forms the DC along with Axin, APC, and CK1. Scaffolding of GSK3β and CK1 via Axin and APC allows for phosphorylation of β-catenin and correspondingly assigns it for ubiquitination [7]. However, it has been documented that phosphorylation of GSK3β actually results in deregulation of the DC and ubiquitination process of β-catenin. Through targeted phosphorylation at specific sites (Ser^9^, Thr^356^, Ser^389^, ect.), GSK3β becomes effectively inhibited [27]. When the DC is destabilized, phosphorylation of β-catenin is halted and downstream genes are transcribed [13]. Research has shown that inhibition of phosphorylation of GSK3β protects functionality of the DC and allows for normal β-catenin phosphorylation. There is a study that revealed numerous allosteric binding sites on GSK3β including sites for ATP, Axin, and the substrate [95]. A recent study tested GSK3β inhibitors targeting these sites but found no definitive suitability from the tested chemicals [96]. This leaves room for future studies testing potential effects of phytochemicals and linkage to allosteric modulation of GSK3β and the Wnt/β-catenin pathway. Notably, the rate-limiting component of the DC is Axin, and is monitored heavily by the Poly (ADP-ribose) polymerases (PARPs) tankyrase [97]. The monitoring of Axin via tankyrase results in the ubiquitination of Axin and eventual accumulation of β-catenin in the intracellular space [97]. However, there is promising research involving small-molecules and possible binding to Axin bypassing tankyrase ubiquitination [98]. The study revealed not only increased GSK3β and subsequent β-catenin phosphorylation, but also noted an increase in RAS/MAPK-mediated K-Ras phosphorylation [98]. This leads to an important discussion about the possible phytochemical/small-molecule involvement with other membrane/nuclear receptors. Possible receptor targets such as the androgen receptor (AR), estrogen receptor (ER), and oxo eicosanoid receptor 1 (OXER1) have shown promising results in binding to phytochemicals discussed in the body of this paper. AR and ER regulate steroid hormone pathways based on two modalities (Genomic/non-genomic). Promising results were discovered for flavonoids like resveratrol, naringin, and quercetin, with binding to orthosteric sites as well as numerous other binding sites [99]. These results further the discussion of the efficacy of phytochemicals in human breast cancer cell lines in numerous pathways outside of the Wnt/β-catenin pathway. Possible pathways for future experiments and studies include the PI3k/Akt pathway, MEK/ERK pathway, STING pathway, and more that have shown linkages to anti-tumorigenesis functionality in breast cancer [100].

## 15. Conclusions and Future Directions

Phytochemicals have the potential to serve as chemopreventive and anti-cancer agents to improve the quality of life and reduce the tumor burden of cancer patients. It is their ability to serve as adjuvants and synergize the outcomes of chemotherapy and radiation therapy makes them an exciting modality to complement traditional therapies. With an appropriate combination of existing treatment approaches, phytochemicals could potentially contribute to reduced side effects and also increase the therapeutic outcome. As reviewed by Kapinova et al. [101], ER-negative breast cancers that are not responsive to selective estrogen receptor modulators such as tamoxifen could be sensitized by phytochemicals and improve the therapeutic outcome when used with other anti-cancer agents. While the results of epidemiological studies are inconclusive on the anti-cancer properties of various phytochemicals, several studies including those from our work highlight the hidden potential of dietary phytochemicals whose biological levels can be achieved by daily consumption of diverse bioactive foods to attain their chemopreventive potential and suppress the development of breast cancer and other malignancies.

To summarize, breast cancers, particularly the aggressive TNBC, have limited therapeutic options. Traditional treatment modalities including chemotherapy, immunotherapy, and radiation therapy are known to have serious side effects and limited benefits against advanced breast cancer. Plant-based phytochemicals have been investigated as alternative treatment options for the treatment and prevention of various cancers including breast cancer. Based on the available data, it is evident that phytochemicals present in a variety of dietary foods along with traditional medicinal usage of plant extracts have a promising role in reducing the burden of various cancers by targeting different pathways involved in the development of cancer. This review article’s main focus is to compile information from existing literature on one such hallmark pathway, the Wnt/β-catenin pathway, targeted by major dietary phytochemicals to overcome the burden of breast cancer development. Numerous studies, including those mentioned in the current review, have demonstrated their usefulness in the prevention and treatment of breast cancer in preclinical studies by modulating the WNT/Beta-Catenin signaling pathway. Despite promising in vitro and in vivo findings presented in this article, there is a need for additional research focusing on the bioavailability studies and determination of the concentrations of these phytochemicals that are essential in providing the therapeutic benefits.

## Figures and Tables

**Table 1:** List of clinical trials.

Author(s)/Principal Investigator	Trial/Paper Name	Phytochemical	Clinical Trial Status	Number of Patients	Intervention	Measures	Outcomes	Clinical Trial ID
Dupoiron et al., 2025 [93]	Evaluating Treatment Preferences and the Efficacy of Capsaicin 179 mg Patch vs. Pregabalin in a Randomized Trial for Postsurgical Neuropathic Pain in Breast Cancer: CAPTRANE	Capsaicin	Completed	140 (70 in group 1 and 70 in group 2)	Group 1: 1–2 capsaicin 8% patches applied to the painful area Group 2: pregabalin 50 mg tablets in 2 doses a day, 100 mg a day, or 150 mg a day	Noninferiority of capsaicin treatment compared to pregabalin treatment for neuropathic pain	Capsaicin treatment was noninferior to daily pregabalin treatment 2 months after surgery	NCT03794388
Saghatelyan et al., 2020 [91]	Efficacy and safety of curcumin in combination with paclitaxel in patients with advanced, metastatic breast cancer: A comparative, randomized, double-blind, placebo-controlled clinical trial.	Curcumin	Completed	150 (75 in Group A and 75 in Group B)	Group A—Curcumin, 300 mg i.v. and paclitaxel, 80 mg/m^2^ BS, i.v., once weekly for 12 weeks. Group B—Placebo i.v. solution and paclitaxel, 80 mg/m^2^ BS, i.v., once weekly for 12 weeks.	Objective response rate (ORR)	ORR was significantly higher in patients treated with curcumin + paclitaxel than placebo + paclitaxel, both after 12 weeks of treatment and 3 months post-treatment.	NCT03072992
	A “Window Trial” on Curcumin, the Active Compound in Turmeric, for Invasive Breast Cancer Primary Tumors	Curcumin	Ongoing	N/A (Not Applicable)	Curcumin, 500 mg twice a day after each meal	Change in tumor proliferation rate	N/A	NCT03980509
Ryan et al., 2013 [94]	Curcumin for Radiation Dermatitis: A Randomized, Double Blind, Placebo-Controlled Clinical Trial of Thirty Breast Cancer Patients	Curcumin	Completed	30 (17 in Group 1 and 18 in Group 2)	Group 1—Curcumin, 2 g three times a day for 4–7 weeks of radiation treatment Group 2—Placebo, 2 g three times a day for 4–7 weeks of radiation treatment	Radiation Dermatitis Severity (RDS)Scale	Patients treated with curcumin had reduced RDS scores compared to patients treated with the placebo.	NCT01042938
Cox et al., 2006 [92]	Influence of Garlic (Allium sativum) on the Pharmacokinetics of Docetaxel	Garlic (Allium sativum)	Completed	11	Docetaxel (30 mg/m^2^) weekly for 3–4 weeks, then 600 mg of garlic tablets twice a day for 12 days	Pharmacokinetics of Docetaxel	Garlic did not significantly affect docetaxel disposition.	NCT00079170
	Dasatinib Combined With Quercetin to Reverse Chemo Resistance in Triple Negative Breast Cancer	Quercetin	Ongoing	N/A	Experimental: quercetin 1000 mg and dasatinib 50 mg weekly	Objective response rate (ORR)	N/A	NCT06355037

## Abbreviations

The following abbreviations are used in this manuscript:

APC	adenomatous polyposis coli
BDMC	bisdemethoxycurcumin
CUR	Curcumin
CSC	Cancer stem cells
CK1	Cysteine kinase 1
DATS	Diallyl trisulfide
DBR	Dihydroberberine
DC	Destruction complex
DKK	Dickkopf
EGCG	Epigallocatechin gallate
EMT	Epithelial–mesenchymal transition
ER	Estrogen receptor
GSK3β	Glycogen synthase kinase 3β
GPCR	G-protein coupled receptor
HER2	Human epidermal growth factor receptor
HPLC	High-performance liquid chromatography
PR	Progesterone receptor
ROS	Reactive oxygen species
TNBC	Triple-negative breast cancer
TCF/LEF	T cell factor/lymphoid enhancer factor
